# Prospective Memory and Positivity Bias in the COVID-19 Health Crisis: The Effects of Aging

**DOI:** 10.3389/fpsyg.2021.666977

**Published:** 2021-07-21

**Authors:** Alaitz Aizpurua, Malen Migueles, Ainara Aranberri

**Affiliations:** Faculty of Pychology, University of the Basque Country UPV/EHU, San Sebastián, Spain

**Keywords:** positivity effect, COVID-19, aging, future events, false memories, positivity bias, personal and social contents

## Abstract

This study aimed to determine whether the observed tendency to remember more positive than negative past events (positivity phenomena) also appears when recalling hypothetical events about the future. In this study, young, middle-aged, and older adults were presented with 28 statements about the future associated with the COVID-19 pandemic, half positive and half negative. In addition, half of these statements were endowed with personal implications while the other half had a more social connotations. Participants rated their agreement/disagreement with each statement and, after a distraction task, they recalled as many statements as possible. There was no difference in the agreement ratings between the three age groups, but the participants agreed with positive statements more than with negative ones and they identified more with statements of social content than of personal content. The younger and older individuals recalled more statements than the middle-aged people. More importantly, older participants recalled more positive than negative statements (positivity effect), and showed a greater tendency to turn negative statements into more positive or neutral ones (positivity bias). These findings showed that the positivity effect occurs in even such complex and situations as the present pandemic, especially in older adults. The results are discussed by reference to the notion of commission errors and false memories resulting from the activation of cognitive biases.

## Introduction

The main research aim of this study is to analyze recall accuracy and transformations in different age groups when recalling hypothetical positive and negative future events linked to the COVID-19 global pandemic. The study was carried out in a state of alarm due to the health crisis of COVID-19, when all the inhabitants of Spain were in lockdown. Being confined at home involves a significant change of routines, especially those linked to work, studies, and leisure (Benke et al., 2020; Vindegaard and Benros, 2020). It implies a loss of freedom and separation of friends and family. The Spanish culture is highly focused on family life and leisure with friends in open spaces, and confinement represents a novel situation that requires an important adaptation process (for a review on the impact of COVID-19 in Spain, see Balluerka et al., 2020; Odriozola-González et al., 2020a,b; Rodríguez-Rey et al., 2020). At the same time, the actual context offers an opportunity to analyze the cognitive processes involved in this emotionally exceptional situation.

Recent studies show that the pandemic is causing feelings of isolation and economic uncertainty in the general population, which are generating higher levels of anxiety and depression and a reduction in the feeling of well-being compared to pre-health-crisis states (Carstensen et al., 2020; Killgore et al., 2020; Odriozola-González et al., 2020b; Vindegaard and Benros, 2020). The numbers are shocking. Results obtained from surveys in China, Spain, Italy, Iran, Turkey, Nepal, and Denmark (Xiong et al., 2020) show that the situation has altered people’s lives, affecting multiple dimensions and generating dramatic increases in stress (8.1–81.9%), anxiety (6.33–50.9%), or depression (14.6–48.3%). The number of suicides associated with joblessness and hopelessness due to an uncertain future in the adult population has increased (Griffiths and Mamun, 2020; Thakur and Jain, 2020). The pandemic has also generated great concern in the university population about the well-being of their family and friends, a negative view of the evolution of their training process, and its impact in the future (Araújo et al., 2020; Odriozola-González et al., 2020a; Zhai and Du, 2020).

All of these aspects may be increased in older people because they are an at-risk population, where contagions leading to death are higher than in the younger population. In addition, the media (TV, press) and social networks at that stage of confinement were filled with news of deaths linked to COVID-19 in nursing homes and hospitals of people who were unaccompanied and without family support in their last moments. While there was still a lack of medical resources and medical instruments for patients with severe symptomatology (e.g., mechanical ventilators), the debate arose as to whether older people should receive such treatments when younger people were in the same situation. The context was significantly more unfavorable for the older population, which could lead to worsening mental health (Armitage and Nellums, 2020; García-Portilla et al., 2020) or suffering from anxiety and depression (e.g., Santini et al., 2020). Cognitive theories of depression indicate that thoughts, inferences, interpretations, and how people attend to and recall fear-related information can be relevant factors to increase depression and anxiety (Mathews et al., 1997; Booth and Sharma, 2020). Taking into account that good emotion regulation requires adequate functioning of the working memory and the inhibitory processes that block access to negative information (Gotlib and Joormann, 2010), older people may be especially vulnerable to mental health problems arising from the pandemic.

Although the global COVID-19 pandemic has paralyzed the world, people’s brain activity has not ceased and continues inexorably to recall their experiences and activities before the pandemic, and to imagine and think about the future. The ability to imagine and plan for the future is a crucial mental process in adaptation, which has been studied in different areas of Cognitive Psychology, especially in episodic future thinking, prospective memory, and mind-wandering (for a recent review, see Kvavilashvili and Rummel, 2020). It is well known that not only does memory recall past experiences, it is also the vehicle that allows us to travel mentally through time to the future (Tulving, 1985, 2005). The projection and mental journey into the future to imagine specific events that may occur, is as frequent as remembering experiences from our past (Finnbogadottir and Berntsen, 2013). Also, thinking about the future activates the same brain areas as remembering past experiences (Addis et al., 2007; Botzung et al., 2008), and both types of episodic thinking have similar characteristics, including sensory and spatial information, and emotion and knowledge about the world (D’Argembeau and Van der Linden, 2004; for a review, see Schacter et al., 2007; Szpunar, 2010). However, there are important differences between thinking about the past and imagining the future. Although both situations involve the recreation and enjoyment of pleasant thoughts and the uncomfortable anticipation of fears and concerns, it has been observed that thoughts about future experiences are more positive than past events (Berntsen and Rubin, 2002; Berntsen and Jacobsen, 2008; García-Bajos et al., 2017; Zaragoza Scherman et al., 2020). Interestingly, according to aging literature, older people tend to remember their past more positively (Kennedy et al., 2004; Schryer and Ross, 2014) and to perceive their future as more idyllic and positive compared to young people (e.g., Berntsen and Rubin, 2002; Burr et al., 2020).

The central concept of this research is individuals’ positivity or our preference for positive information as opposed to negative information when performing attention and memory tasks; this preference is enhanced in older adults, a phenomenon known as the positivity effect (Charles et al., 2003; Carstensen and Mikels, 2005; Mather and Carstensen, 2005; Reed and Carstensen, 2012; Schryer and Ross, 2014; García-Bajos et al., 2017). Thus, positivity effect means that, compared to young people, older people react less to negative situations and preferentially attend to and recall emotionally meaningful and positive stimuli (Reed et al., 2014). This positivity effect has been observed with a multitude of materials such as scenes, drawings, and faces (e.g., Charles et al., 2003; Mather and Carstensen, 2005; Reed et al., 2014; Mammarella et al., 2016), words (Kensinger, 2008; Hamilton and Allard, 2020), or autobiographical experiences of the past and recreations of the future (Berntsen and Jacobsen, 2008; Gallo et al., 2011; Cole et al., 2016; García-Bajos et al., 2017). Older people not only show a greater preference for the positive than young people, but they also generate false memories or transform and modify negative content to make it more positive and, thus, achieve greater consistency with their emotional goals and motivations, and higher emotion regulation and well-being (Charles et al., 2003; Carstensen et al., 2020; Zaragoza Scherman et al., 2020). Although the positivity effect is robust and consistent, as shown by the meta-analysis of 100 studies on the subject by Reed et al. (2014), some contrasting results has shown practically no differences between young and older adults (e.g., Kensinger et al., 2002; Grühn et al., 2005). A less-studied aspect is whether middle-aged adults liken their performance to that of young participants or are closer to that of older people (Carstensen and DeLiema, 2018).

Various theories have been proposed to explain the positivity effect. Some theories posit that age-related advantages reflect the avoidance of stressors (Charles, 2010), whereas others maintain that the advantages of age are driven by motivational shifts that direct cognitive and behavioral resources toward positive and meaningful aspects of life (Carstensen et al., 2020). The Socio-emotional Selectivity Theory (SST; Carstensen et al., 1999; Carstensen, 2006) emphasizes an increase with age to the accessibility of positive information. A person’s priorities and motivations change with age. The fragility of life and the reduction of life expectancy lead them to prioritize objectives, ideas, and content that afford them general satisfaction and that are pleasurable and rewarding. Other theories underscore older people’s difficulty to recreate and imagine the future and argue that generating and processing positive future events requires less cognitive effort and less time than negative events (Newby-Clark and Ross, 2003; Schacter et al., 2008; Berntsen and Bohn, 2010), mainly because negative content is more complex to process than positive content (Labouvie-Vief et al., 2010). Finally, it is also proposed that older people focus on emotion regulation by implementing their cognitive control resources, such as activating inhibitory resource to block access to negative information (García-Bajos and Migueles, 2017; Giebl et al., 2016; Marsh et al., 2019). That is, cognitive abilities and motivation contribute to the positivity effect.

This study has three priority objectives. First, to analyze in three age groups possible differences between the recall of hypothetical future negative thoughts related to the threats and repercussions of COVID-19 and positive thoughts for the future, desires, and plans after the pandemic. It could be considered that the current situation leads to focusing on COVID-19-related sources of fear (Mathews et al., 1997; Booth and Sharma, 2020) and that this, in turn, can lead to a state of mood-congruent retrieval, focusing recall on negative content (for a review, see: Blaney, 1986). However, the literature also indicates that to increase the sense of well-being and reduce stress and anxiety, people prefer to codify and remember positive aspects, showing a positivity phenomenon. Thus, we expect that participants in our study will show a tendency to process and remember positive statements better than negative ones; and we anticipate that this trend will be more pronounced in older people than in young adults, that is, a positivity effect. Second, we shall examine the transformations, biases, errors, and false memories that emerge to face adversity in individuals of different age groups, and we expect to see a greater positive bias in older than in young people. This finding was hypothesized because, as with other types of false memories, older people tend to use the cognitive and attentional resources available to them to adjust their thoughts to their previous knowledge (e.g., Schacter et al., 1997), and to regulate their emotional state, in this case, by imagining the events of their own future, adapting it to their desires and personal expectations. Thirdly and lastly, we analyze the effects on recall of the social or personal nature of the thoughts about the hypothetical future. It has been observed that the recreation of the future is more likely to be performed in the third person rather than from a first-person perspective (D’Argembeau and Van der Linden, 2004, 2006). In addition, in the current context of pandemic, social content can be perceived as high-value information or more important than personal content (Hargis and Castel, 2017). Therefore, we expect a better recall of social than of personal imagined future events. However, little is known about the effect of the social or personal perception of future experiences, and whether one’s perspective interacts with the positive or negative valence of thoughts and/or with the age of the individuals who recall those thoughts.

In regard to the emotional regulation, although age-related biological, psychological, and socio-economic factors are not favorable for older people, the literature provides us with abundant data indicating that older people’s emotional experiences are more stable and positive than younger people’s, and they also show a greater sense of well-being (Carstensen et al., 2000; Carstensen, 2006; Stone et al., 2010; Burr et al., 2020). This may be due, at least in part, to the fact that older people have more pronounced mechanisms than young people to adapt to adversity, sources of stress, and emotionally negative events. For example, Carstensen et al. (2020) interviewed people between the age of 18 and 76 in the middle of the COVID-19 pandemic, assessing the frequency and intensity of a range of positive and negative emotions, and the subjectively perceived risk of contagion and complications from the virus. They observed that older people showed relatively greater emotional well-being than young people. In other words, older people appear to have greater resilience and mechanisms to regulate their emotions and deal with adverse situations positively (Fontes and Neri, 2015; Silva Junior et al., 2019). One of these mechanisms is the positivity bias.

Positivity bias in autobiographical memory and episodic future thinking is considered important in mental wellbeing, as a cognitive strategy to reduce stress and depression. When attempting to remember positive and negative thoughts linked to the pandemic, one’s memory works to find a way out of the situation. Memory uses adaptive cognitive processes, which reconstruct reality using preexisting knowledge, beliefs, expectations, and desires, and generate errors and distortions (Schacter, 1999, 2021; Schacter et al., 2011); one of these distortions is the positivity bias, which make one less vulnerable to emotional disorders (such as depression and anxiety), and help improve mood.

To summarize, the main objective of this study is to examine in three age groups (young, middle-aged and older adults) how the situation of pandemic and confinement affects the memory of positive and negative thoughts of the future linked to COVID-19. We are interested in determining if the situation of stress, fear and worry that we are experiencing induces us to remember more content congruent with that depressed mood or if, on the contrary, a positive effect appears and we remember more positive content. This tendency to remember more positive than negative content may be accentuated in older people (positivity effect), and possibly so does the tendency to turn negative statements into more positive or neutral ones (positivity bias), both understood as cognitive strategies to achieve emotional regulation and feelings of well-being; *a priority* as we get older. Analyzing these aspects and knowing how middle-aged people behave are priority objectives of this study.

## Materials and Methods

### Participants

Participants were 33 young adults (*M* age = 20.33, *SD* = 1.93; range: 19–25 years), 23 middle-aged adults (*M* age = 42.48, *SD* = 7.29; range: 28–54 years), and 23 older adults (*M* age = 64.27, *SD* = 5.81; range 55–77 years). The young participants were students of different degrees at the University of the Basque Country (UPV/EHU), and the older participants came from cultural groups or were undergraduate students at the University of the Basque Country (UPV/EHU) who pursued a humanities career for older people called Experience Classrooms. An *a priori* power analysis was conducted with G^∗^Power (Faul et al., 2009) to determine the sample size required to achieve a medium effect size of *F* = 0.25, with a significance level of α = 0.05, and statistical power of 0.80. A 3 (Group: Young vs. Middle-aged vs. Older) × 2 (Valence: Positive vs. Negative) × 2 (Nature: Personal vs. Social) mixed factorial design with group as a between-participants variable, and the emotional valence and nature of the statements as within-participants variables require a minimum sample of 17 for each age group. In this study, at least 22 participants were included in each age group.

### Materials and Procedure

This study was carried out following the American Psychological Association standards for the ethical treatment of participants, the Declaration of Helsinki, and was approved by the Ethics Committee of the University of the Basque Country (UPV/EHU). Participants were first informed that the experiment dealt with the positive and negative nature of thoughts about the future linked to the COVID-19 pandemic. Because the alarm status had been decreed in Spain, and the entire population was confined without the possibility of leaving their homes, this experiment was conducted online through Google forms. The survey was disseminated through the student council, coordinators, and undergraduate delegates, and the university’s website, which opened a space for studies linked to COVID-19. In the form, participants were asked: “In the stage of lockdown and pandemic due to COVID-19, one thinks about the future and imagines positive experiences and facts, but negative fears and experiences also come to mind about what could happen to us in the near or distant future.” They were informed that they would be presented with a total of 32 statements or thoughts about the future and they should rate their level of agreement with each of those thoughts. Of these statements, 28 were experimental, 14 positive and 14 negative, and the other 4 were used to control the primacy and recency effects and were not included in subsequent analyses.

Each participant received positive and negative thoughts randomly (with no more than two positive or negative statements in a row), but they were not instructed that they would subsequently be requested to perform a recall task. The statements were drawn from future estimates from the news, newspapers, and social media’s concerns and aspirations. To select the statements, 21 adults who did not participate in the study evaluated the statements in two dimensions: valence (positive or negative) and nature (personal or social), and we chose those that obtained clearly defined scores (more than 70% agreement)^1^. Half of the statements were positive (e.g., “I think we will be strengthened by this pandemic”), and the rest were negative (e.g., “I think this virus will mutate and we won’t be able to beat it”). Also, half of the positive and negative ideas were personal (e.g., “This pandemic helps me know myself better”) or had a more social connotation (e.g., “Popular concerts and festivals won’t come back”). After the participants had received the instructions and agreed to participate in the study, a statement and a scale ranging from 1 (*totally disagree*) to 7 (*totally agree*) appeared on the screen to rate their level of agreement.

The participants worked at their own pace and after rating all the sentences, a distracting task consisting of writing words that started with S for 3 min appeared. After the distracting task, a free recall task was administered. Participants were encouraged to write as many of the previously presented thoughts about the future as possible, in any order. This free recall task has the additional advantage of revealing participants’ strategies to organize the material. The experimental phase lasted approximately 10–15 min.

### Design

The present study employed a 3 (Group: Young vs. Middle-aged vs. Older) × 2 (Valence: Positive vs. Negative) × 2 (Nature: Personal vs. Social) mixed factorial design with group as a between-participants variable, and the emotional valence and nature of the statements as within-participants variables. Correct recall and errors were measured for the positive and negative contents produced by each participant.

## Results

### Rating Thoughts About the Future

Participants were asked to rate their level of agreement with each of the statements about the near future. The agreement level was higher than the average value of 3.5, both in general and in all the age groups (all *p*s < 0.001). In order to estimate the internal consistency of the material employed, Cronbach’s Alpha was calculated for the agreement with the positive and negative items in the sample studied. These two values indicate a good level of reliability for both the positive (α = 0.77) and negative (α = 0.73) statements.

In order to analyze the ratings given by the participants, a 3 (Group: Young vs. Middle-aged vs. Older) × 2 (Valence: Positive vs. Negative) × 2 (Nature: Personal vs. Social) ANOVA was conducted. The analyses indicated that the Group factor was non-significant in the ratings, *F* < 1. That is, there were no differences between young, middle-aged, and older adults in their level of agreement with the statements (see Table 1).

**TABLE 1:** Mean proportion and standard deviations (in parentheses) in total agreement with the statements depending on their valence (positive, negative) and nature (personal, social), in the different age groups.

**Group**	**Agreement**	**Positive**	**Negative**	**Personal**	**Social**
Young	4.18 (0.43)	4.65 (0.66)	3.64 (0.75)	4.14 (0.33)	4.13 (0.64)
Middle-aged	4.01 (0.65)	4.55 (0.88)	3.37 (0.67)	3.64 (0.66)	4.27 (0.69)
Older	4.20 (0.78)	4.51 (0.98)	3.81 (0.86)	4.01 (0.86)	4.32 (0.77)
Total	4.14 (0.61)	4.58 (0.82)	3.61 (0.77)	3.96 (0.65)	4.23 (0.69)

The Valence variable was significant, *F*(1, 76) = 65.33, *p* < 0.001, η*_*p*_*^2^ = 0.462, indicating a higher level of general agreement with positive than with negative thoughts (*M* = 4.58, *SD* = 0.82, vs. *M* = 3.61, *SD* = 0.77). The effects of the variable Nature, *F*(2, 76) = 26.63, *p* < 0.001, η*_*p*_*^2^ = 0.260, were also significant, as higher agreement was observed for the social statements than for the personal ones (*M* = 4.23, *SD* = 0.69, vs. *M* = 3.96, *SD* = 0.65). The significant Group × Nature interaction, *F*(1, 76) = 9.98, *p* < 0.001, η*_*p*_*^2^ = 0.208, showed that the level of agreement was higher for social statements than for personal ones in middle-aged adults, *t*(22) = 6.21, *p* < 0.001, and older adults, *t*(22) = 3.59, *p* = 0.002, but not in young adults, who rated the dimensions of both statements equally. The Valence x Nature interaction was also significant, *F*(1, 76) = 50.76, *p* < 0.001, η*_*p*_*^2^ = 0.400, indicating a greater level of agreement with positive statements of a social nature (*M* = 4.50, *SD* = 0.93), followed by personal positive statements (*M* = 4.66, *SD* = 0.86), negative social statements (*M* = 3.96, *SD* = 0.92), and finally, negative statements of a personal nature (*M* = 3.26, *SD* = 0.84), with all the differences between them statistically significant, *p* < 0.05. To some extent, these ratings of the level of agreement show a preference for positive aspects when individuals imagine their future, a consistent result with previous outcomes related to positivity.

### Recall of Thoughts About the Future

In the recall task, those participants who recalled two or less correct statements were discarded (this happened with two participants in each age group). The rating criteria to correct free-recall task were strict. To consider a sentence as correct, a literal replication of the original statements was not required, but the preservation of the gist (i.e., the defining content of the sentence) or relevant details were needed, because it is known that memory is of a reconstructive nature (for example, “This coronavirus is a bioweapon created in the laboratory” was considered correct as the recall of the sentence “I believe that the coronavirus is part of a biological war”). Free recall was scored by two judges assigning one point for every correctly recalled sentence. The very few discrepancies were resolved by a third independent judge blind to the experimental conditions.

In order to analyze the thoughts recalled by the participants, a 3 (Group: Young vs. Middle-aged vs. Older) × 2 (Valence: Positive vs. Negative) × 2 (Nature: Personal vs. Social) ANOVA was conducted (see Table 2). The effects of the Group factor were significant, *F*(2, 76) = 5.50, *p* = 0.006, η*_*p*_*^2^ = 0.013. Although there were no differences between young and old adults, young people remembered a higher proportion of thoughts about the future than middle-aged adults, *t*(55) = 3.21, *p* = 0.002, *d* = 0.43.

**TABLE 2:** Mean proportion (SD) of correct recall depending on their valence (positive and negative) and nature (personal, social), in the different age groups.

**Group**	**Recall**	**Positive**	**Negative**	**Personal**	**Social**
Young	0.30 (0.14)	0.30 (0.16)	0.31 (0.14)	0.29 (0.17)	0.31 (0.14)
Middle-aged	0.19 (0.11)	0.19 (0.12)	0.20 (0.15)	0.19 (0.14)	0.20 (0.13)
Older	0.25 (0.12)	0.31 (0.16)	0.20 (0.11)	0.23 (0.13)	0.28 (0.18)
Total	0.26 (0.13)	0.27 (0.16)	0.25 (0.15)	0.24 (0.15)	0.27 (0.16)

The variable Nature was non-significant, *F* < 1. There were no differences between the recall proportions of personal and social thoughts (*M* = 0.24, *SD* = 0.15 vs. *M* = 0.27, *SD* = 0.16) in the total sample.

**FIGURE 1:**
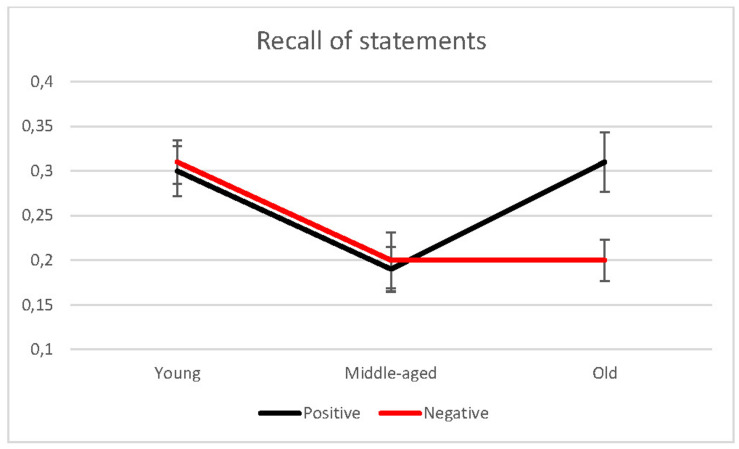
Recall of positive and negative statements in young, middle-aged, and older adults.

The Valence variable was also non-significant, *F*(1, 76) = 3.51, *p* = 0.065, η*_*p*_*^2^ = 0.044, revealing no statistically significant differences in the total sample between the recall rates of positive and negative thoughts (*M* = 0.27, *SD* = 0.16 vs. *M* = 0.25, *SD* = 0.15). However, the effects of the Group × Valence interaction were significant, *F*(2, 76) = 6.11, *p* = 0.003, η*_*p*_*^2^ = 0.014, indicating that, unlike the other two age groups, older people were influenced by the valence of thoughts, with a greater recall of positive thoughts (*M* = 0.31, *SD* = 0.16) than of negative ones (*M* = 0.20, *SD* = 0.11), *t*(22) = 3.50, *p* = 0.002 (see Figure 1). That is, as predicted by the positivity effect, older people showed a preference for positive content, recalling the same number or more of positive thoughts and the same number or fewer negative thoughts than young and middle-aged adults. In other words, for positive thoughts, middle-aged adults recalled a lower proportion than young adults, *t*(54) = 2.79, *p* = 0.007, *d* = 0.38, and older adults, *t*(44) = 2.85, *p* = 0.007, whereas, in the case of negative thoughts, young adults recalled significantly more thoughts than older people, *t*(54) = 3.13, *p* = 0.003, *d* = 0.43, and middle-aged adults, *t*(54) = 2.83, *p* = 0.006. There were no more significant interactions between the variables. In addition, correlational analyses between rating and recall of thoughts were conducted but the results were not significant, *r* (79) = 0.04, *p* = 0.718.

We also examined the clustering of positive and negative statements of the to be remembered material. Clustering refers to the tendency for items to take place next to one another in time. To quantify clustering we applied the Adjusted Ratio of Clustering (ARC; Roenker et al., 1971; Senkova and Otani, 2012), in which chance clustering is set at 0, perfect clustering at 1, and negative scores indicate clustering below chance. We calculated this measure for both, the material administered in the encoding phase, and the final free recall performance. The ARC value was negative (–0.62) for the positive and negative statements presented to the participants, showing that statements were interleaved during the encoding phase. For the recall performance, ARC value in the total sample was also negative (–0.14), without statistically significant differences between young (–0.12), middle.age (–0.10) and older adults (–0.25), *F*(2, 65) = 0.60, *p* < 0.55, η*_*p*_*^2^ = 0.014. Therefore, the participants in the 3 age-groups interleaved positive and negative thoughts in the final recall task much the same way as they were clustered within the material presented in the encoding phase. In addition, clustering of the positive or negative statements in final recall cannot explain the positivity bias, because the ARC scores were below zero for positive and negative statements. We also analyzed the number of repetitions of the positive and negative statements in the total sample in the final recall task. The only interesting result was that young participants (*M* = 1.48, *SD* = 1.4) were more likely to repeat negative statements than older adults (*M* = 0.89, *SD* = 0.89), although this was only a tendency, *t*(46) = 1.77, *p* = 0.084). The correlations between ARC and number of repetitions (both positive and negative statements) were not statistically significant.

### Transformations and Biases

When recalling thoughts about the future, the participants sometimes modified their positive or negative valence. Analyzing these changes of valence or transformations allowed us to examine the memory biases during the personal elaboration and recovery of the previously presented material. Four types of transformations were classified: (1) initially negative statements transformed into neutral (2) or positive statements, (3) originally positive statements modified to neutral (4) or negative statements.

In order to analyze the transformations made by the participants, a 3 (Group: Young vs. Middle-aged vs. Older) × 2 (Nature: Personal vs. Social) ANOVA was conducted. The first two types of transformations (see Table 3) were considered a consequence of the positivity bias, because the participant positivized the statements by removing the negative connotation (e.g., *I talk a lot about this subject, I am obsessed and I do not cease looking for information → I have tried to obtain information about it. This will lead to a negative change at the social and political level → There will be political-social change*), or by transforming the idea into something positive (e.g., *Mass concerts will not come back, nor will parties nor the great stadiums → I think partying is something that will soon come back. I think that the coronavirus is part of a biological warfare → I don’t think the virus is an invention*).

**TABLE 3:** Mean proportion (SD) of valence changes from initially negative to neutral or positive statements, for personal and social claims in young, middle-aged, and older adults.

	**From negative to neutral**			**From negative to positive**		
**Group**	**Change**	**Personal**	**Social**	**Change**	**Personal**	**Social**
Young	0 (0)	0 (0)	0 (0)	0.09 (0.29)	0 (0)	0.18 (0.58)
Middle-aged	0.07 (0.17)	0 (0)	0.13 (0.34)	0.02 (0.10)	0 (0)	0.04 (0.21)
Older	0.15 (0.24)	0.09 (0.29)	0.22 (0.42)	0.04 (0.14)	0 (0)	0.09 (0.29)
Total	0.06 (0.17)	0.03 (0.16)	0.10 (0.30)	0.06 (0.21)	0 (0)	0.11 (0.42)

In transformations from negative to neutral, the Group factor was significant, *F*(1, 76) = 6.38, *p* = 0.003; η*_*p*_*^2^ = 0.144. Without any differences between them, both older adults (*M* = 0.15, *SD* = 0.24) and middle-aged adults (*M* = 0.07, *SD* = 0.17) had a greater tendency to positivize initially negative phrases than did young adults, who did not produce any examples. The Nature variable also had significant effects, *F*(1, 76) = 4.79, *p* = 0.032; η*_*p*_*^2^ = 0.059, indicating that social statements (*M* = 0.10, *SD* = 0.30) were generally more positivized than personal ones (*M* = 0.03, *SD* = 0.16). The Group x Nature interaction was non-significant. Only the variable Nature, *F*(1, 76) = 4.62, *p* = 0.035; η*_*p*_*^2^ = 0.057, was significant in the negative to positive transformations because these changes were observed only for social statements (i.e., no examples of negative-to-positive transformations were observed for statements with a personal connotation). The Group × Nature interaction was non-significant.

The other two types of transformations, that is, initially positive thoughts that were transformed into neutral or negative ones, were very scarce (see Table 4). Participants transformed initially positive to neutral phrases (e.g., *I have good prospects for the future → This will influence my future. Although we will need time, we will travel again* → *The way we travel will change*) and they also negativized originally positive thoughts to negative ones (e.g., *The crisis is bringing out our best → I don’t think we will be better people after the crisis. This confinement has allowed me to meet my neighbors and have new friends → I think this has not brought me any closer to my family or neighbors.*).

**TABLE 4:** Mean proportion (SD) of changes in valence from initially positive to neutral or negative statements, for personal and social statements in young, middle-aged, and older adults.

	**From positive to neutral**			**From positive to negative**		
**Group**	**Change**	**Personal**	**Social**	**Change**	**Personal**	**Social**
Young	0.03 (0.12)	0.06 (0.24)	0 (0)	0.11 (0.30)	0.03 (0.17)	0.18 (0.58)
Middleage	0.04 (0.14)	0 (0)	0.09 (0.29)	0.04 (0.14)	0 (0)	0.09 (0.29)
Old	0.02 (0.10)	0 (0)	0.04 (0.21)	0.09 (0.19)	0.04 (0.21)	0.13 (0.34)
Total	0.03 (0.12)	0.03 (0.16)	0.04 (0.19)	0.08 (0.23)	0.03 (0.16)	0.14 (0.45)

In the first type of negativizations, there were no significant effects of the variables, whereas in the case of positive statements transformed into negative ones, only the variable nature was significant, *F*(1, 76) = 3.84, *p* = 0.054; η*_*p*_*^2^ = 0.048, revealing that these changes were observed to a greater extent for social statements than for statements with a personal connotation.

## Discussion

The central objective of this study was to examine the recall of positive and negative thoughts about the future linked to the global COVID-19 pandemic, and to examine the biases, errors, and distortions that occur in the recall of emotional information, especially positive biases in young, middle-aged, and older adults. Although the participants knew they were in an experiment on aspects related to COVID-19, they believed that their task was to rate their level of agreement and disagreement with the ideas and thoughts proposed about the future, and did not expect the task of recalling these contents. It is well known that incidental learning leads to worse performance than intentional learning, but it has also been observed that the effects of positivity are accentuated when participants are free to remember and are not subject to restrictions on how to organize their recall (Reed et al., 2014; García-Bajos et al., 2017). For example, positivity is not evident when the instructions request participants to encode the stimulus valence (Kensinger et al., 2002) or to accurately recall all the information (Grühn et al., 2005).

This study was carried out in the midst of the alarm state, when the population had already been confined for more than 2 weeks, and the streets were deserted and people could only go outside to acquire essential products. With the media and social networks full of bad news, the question arises as to how proposed hypothetical future events will be recalled. It is reasonable to think that people’s thoughts and inferences will lead them to focus on the sources of fear implied by COVID-19 (Mathews et al., 1997; Booth and Sharma, 2020) producing a mood-congruent retrieval, that is, focusing recall on events associated with a negative emotional state. There is actually a good deal of evidence for this “mood-congruency” effect for a variety of cognitive processes, including attention and perception, judgment, and various types of recall and recognition procedures (for reviews, see Blaney, 1986; Siemer, 2005; Koster et al., 2010; Sasa, 2013). However, the theory of mood congruence is not met because, even if there was fear of contagion and the unknown consequences of COVID-19 at that particular moment of confinement, there was also expectation, novelty, new activities that were being incorporated into people’s routine and thoughts about the positive aspects that this pandemic could imply (e.g., becoming more humane, more supportive or empathetic, uniting more as a society in the face of adversity. and/or improving awareness of the environment). Our data show a phenomenon of positivity. On the one hand, in the subjective ratings of the contents where the participants, regardless of age, agreed more with the positive than the negative content, and, on the other hand, in the recall task, where, despite the health crisis, they retrieved positive and negative thoughts to the same extent. Both effects may reflect a mood-regulation strategy. Enhancing the idea of positivizing the situation, the participants even biased their recall by transforming content initially presented as negative into more neutral or positive content.

Also in this study, we observed that the positivity phenomenon is enhanced in older people, giving rise to the positivity effect observed in many previous studies (e.g., Carstensen and DeLiema, 2018; Gallo et al., 2011; García-Bajos et al., 2017). Although globally, there are no differences between the recall of positive and negative facts, the interaction between the main variables Group and emotional Valence shows that older people have a greater recall of positive imagined future events and greater resistance to recalling negative facts (Charles et al., 2003), whereas young people present a better performance than the older and middle-aged people in the recall of negative content. At the same time, older and middle-aged adults tend to transform negative statements into neutral ones, eliminating terms that give a negative connotation to the idea or changing the statements to more pleasant possibilities. In other words, older people distance themselves from the negative possibilities and consequences of the future, thinking about it in a more generic, less specific, or more semantic way (Devitt and Schacter, 2020), thus achieving the goal of regulating mood by decreasing negative emotions and increasing positive ones (Rusting and DeHart, 2000). Similar results have been observed for past choices and autobiographical information, where older people show more emotionally gratifying memory distortion than young adults (Mather and Carstensen, 2005). Our data further indicate that this positive bias is also characteristic of middle-aged people, an age group little analyzed in the literature on cognition and memory in general and on the positivity effect in particular.

To what is this positivity effect due? Various theories have been proposed to explain the effect of positivity. Some theories posit that age-related advantages reflect the avoidance of stressors (Charles, 2010) but this is difficult to accept in the current pandemic situation and its effects. Our data are more consistent with the Socio-emotional Selectivity Theory (SST; Carstensen et al., 1999; see also Carstensen and Mikels, 2005; Carstensen et al., 2006; Reed and Carstensen, 2012). The SST is a life-span theory of motivation, which proposes that, because of the realization that the time left to live is growing shorter, older adults are more likely to prioritize their balance and emotional well-being. Priorities change with age, and a preference for the positive emerges. The SST posits that older adults deploy cognitive control mechanisms to suppress negative stimuli and to seek out positive, emotionally rewarding information. Although at the cognitive level, older people generally show deficits in resource availability, they use their resources to enhance emotion regulation, perhaps using their limited resources to block or inhibit negative thoughts and activate positive ones (Giebl et al., 2016; García-Bajos and Migueles, 2017; García-Bajos et al., 2017; Marsh et al., 2019). Although not as accentuated as in older people, the fact that middle-aged participants also show the positivity effect suggests that the effect is not due to a malfunction of the amygdala that reduces neural and affective responses to negative stimuli (Reed and Carstensen, 2012) or to the fact that the processing of negative content is more complex and cognitively more demanding (Labouvie-Vief et al., 2010). The results rather suggest that cognitive abilities and motivation both contribute to the outcomes obtained from improved emotion regulation as people get older (Mather and Carstensen, 2005).

An interesting aspect is the performance of the group of young people, university students concerned about their future training and work. Although the real impact of COVID-19 on students’ education and mental health is still unknown (Araújo et al., 2020; Odriozola-González et al., 2020a; Sahu, 2020), psychological symptoms are common in the university population (Bayram and Bilgel, 2008; Auerbach et al., 2016). A study conducted by Odriozola-González et al. (2020a) analyzed the impact of COVID-19 during the first weeks of confinement in Spanish university students. They applied an online survey to 2530 students and observed that moderate to extremely severe scores of anxiety (21.34%), depression (34.19%), and stress (28.14%), and a total of 50.43% of the respondents presented a moderate to severe impact of the outbreak. In our study, although the young students agreed more with the positive than the negative statements, they showed no positivity effect. In their performance, there were no differences between the recall of positive and negative content, but they recalled significantly more negative content than older and middle-aged adults. In other words, they showed less resilience, which reflected their concern about the potentially negative impact on their academic progress.

Finally, concerning the effects of the social or personal nature of the thoughts about the future, our results have shown that, although young people provided similar ratings for both types of content, older and middle-aged participants rated social thoughts as closer to their way of thinking than personal thoughts, an effect we could call sociability. As for memory, contrary to our expectations of a trend of a greater recall of social than of personal content because it is easier to imagine and recreate the future in third-person than in the first-person (D’Argembeau and Van der Linden, 2004, 2006), there were no differences in recall between the two types of content, which was equivalent in the participants of all age groups. Although young people recruit effective encoding strategies to remember a large amount of information regardless of its nature, older people perceived and categorized the emotional content of a social nature as more relevant. It has been observed that emotional content rated as important reduces the differences between the recall of young and old people (Denburg et al., 2003; Mather, 2004; Spaniol et al., 2008). In general, social statements were more positivized than personal ones, that is, the positivity bias was greater for statements about society as a whole than for statements about particular individuals, and it would be relevant to analyze these aspects concerning recall and false memories in the future.

Although this study has the limitations of having been carried out online, which leads to a reduction of situational control, it has the advantage of immediacy and of being able to rigorously examine the recall of content and thoughts about the future after a pandemic that is changing the world. Future research should examine whether executive functions also influence the accuracy of recalling content about the future. It would also be interesting to analyze individual differences (especially in the older group) in positivity bias because there may be great variability, as with other types of false memories (Gerrie and Garry, 2007; St Jacques et al., 2015; Greene and Murphy, 2020). The investigation of the particularities of cognitive functioning and memory of middle-aged adults is relevant from a cognitive research perspective.

In short, this study increases our understanding not only of the impact of aging on the memory of imagined positive and negative future events and their transformations and modifications but also middle-aged adults’ recall of episodic future events and false memories. The errors, distortions, and transformations observed in this study do not have the numerical scope of the errors observed with the DRM paradigm (Roediger and McDermott, 1995), where a set of associated words (e.g., pin, puncture, pain, syringe) induce the recall of an unpresented word (needle), or the applied impact in the judicial sphere such as the post-event information procedure, where suggested information is introduced in the recall (Loftus, 1991, 2003); however, they show the mind’s ability to spontaneously transform content to make it kinder, more positive, and to help people to adapt to adversity, reduce anxiety and depression, foster resilience, and contribute to feelings of well-being. The positivity bias shows the adaptive value of the memory (e.g., Schacter, 1999, 2021; Schacter et al., 2011), which does not disappear and may even be enhanced in crises, such as the one we are currently experiencing.

## Data Availability Statement

The original contributions presented in the study are included in the article/supplementary material, further inquiries can be directed to the corresponding author/s.

## Ethics Statement

This study was carried out in accordance with the American Psychological Association standards for ethical treatment of participants, the Declaration of Helsinki, and was approved by the Ethics Committee of the University of the Basque Country UPV/EHU (Ref. M10_2016_052). Participants were informed that the experiment dealt with the positive and negative nature of thoughts about the future linked to the COVID-19 pandemic. Participation was voluntary, and consent was presumed by the completion of the survey. Written informed consent was not provided because this experiment was conducted online through Google forms, because the alarm status had been decreed in Spain, and the entire population was confined without the possibility of leaving their homes.

## Author Contributions

MM and AAi conceived, designed, prepared the materials for the experiment, performed the experiment, collected the data, scored the tasks, and wrote the manuscript. AAi analyzed the data. AAr helped re-analyzing the data and reviewed the final version of the manuscript. All authors critically reviewed the manuscript for important intellectual content, approved the manuscript for publication, and agreed to be accountable for all aspects of the work in ensuring that issues related to the accuracy or integrity of any part of the work are appropriately investigated and resolved. All authors contributed to the project of this research.

## Conflict of Interest

The authors declare that the research was conducted in the absence of any commercial or financial relationships that could be construed as a potential conflict of interest.

## Footnotes

1 The participants who valued the sentences were representative of the three age groups and similar to the total sample examined in the study; specifically, they were 6 young adults (*M* age = 20.33, *SD* = 1.93), 8 middle-aged (*M* age = 42.48, *SD* = 7.29), and 7 old adults (*M* age = 63.87, *SD* = 5.99), and 13 women and 8 men.
